# Re-response to tolvaptan after furosemide dose reduction in a patient with refractory ascites

**DOI:** 10.1007/s12328-014-0545-8

**Published:** 2014-12-05

**Authors:** Atsushi Goto, Shuji Terai, Munetaka Nakamura, Masaharu Matsumoto, Isao Sakaida

**Affiliations:** 1 Department of Gastroenterology and Hepatology, Hagi Civil Hospital, Tsubaki 3460-3, Hagi, Yamaguchi 758-0061 Japan; 2 Department of Gastroenterology and Hepatology, Yamaguchi University Graduate School of Medicine, Minami-Kogushi 1-1-1, Ube, Yamaguchi 755-8505 Japan

**Keywords:** Tolvaptan, Vasopressin V2-receptor antagonist, Liver cirrhosis, Refractory ascites, Urine osmolality

## Abstract

Tolvaptan is a new drug used for treating ascites induced by liver cirrhosis, and it is covered by health insurance in Japan. In the present report, we describe the case of a 74-year-old man with type C liver cirrhosis and refractory ascites. He was receiving furosemide and spironolactone daily, but still required repeat puncture for ascites removal. Administration of tolvaptan (3.75 mg/day) was started in addition to his existing medications, and was subsequently increased to 7.5 mg/day. However, after 2 months, the ascites again exacerbated. Nevertheless, after we discontinued the administration of furosemide, the tolvaptan became effective. This may be because furosemide administration decreases urine osmolality, resulting in a non-response to tolvaptan.

## Introduction

The selective vasopressin V2-receptor antagonist tolvaptan is a newly introduced diuretic, and has a different mechanism as compared with conventional loop diuretics [1]; in Japan, it has been used for fluid retention in heart failure patients since 2010. In 2013, it was approved for treating fluid retention associated with liver cirrhosis (in doses up to 7.5 mg/day). Tolvaptan is an option for managing body fluid in liver cirrhosis patients who have been managed by furosemide and/or spironolactone, and it has great potential for treating patients with a refractory condition. However, many issues remain unclear, including the combined method for using it with existing drugs, dosing period, and prerequisites for obtaining favorable outcomes. In the present report, we describe a case of refractory ascites associated with type C liver cirrhosis wherein a complete response was achieved immediately after administering tolvaptan; however, the ascites exacerbated after 2 months, and the drug’s diuretic effect was restored by discontinuing furosemide.

## Case report

A 74-year-old man presented to our hospital with abdominal pain and bloating since December 2013. He had undergone craniotomy for a brain tumor approximately 30 years ago. He had no history of alcohol consumption or cigarette smoking, and his family history was not significant. Since 2001, he had been managed regularly for type C liver cirrhosis. He did not have a treatment history of interferon use. He had undergone sclerotherapy for esophageal varices in 2001 and hepatic arterial chemoembolization in 2004, and did not have any recurrences. However, he continued to receive oral furosemide (20 mg) and spironolactone (50 mg) daily for ascites retention. Although he maintained a body weight of 60 kg and an abdominal circumference of 85 cm, his body weight and abdominal circumference rapidly increased to 70 kg and 100 cm, respectively. Ultrasound confirmed the presence of increased ascites; thus, the dose of furosemide was increased to 30 mg/day, and puncture was repeated for ascites removal. Since there was no improvement, he was hospitalized in January 2014.

On admission, the following physical findings were noted: height, 168 cm; body weight, 69 kg; abdominal circumference, 100 cm; consciousness, lucid; body temperature, 35.5 °C; blood pressure, 137/85 mmHg; pulse, 80 beats/min (regular). The palpebral conjunctiva showed no sign of anemia. The bulbar conjunctiva had no yellow staining. The chest findings were normal with no evidence of heart murmur or spider angioma. The abdomen was swollen, severely distended, and wave-palpable. He had spontaneous abdominal pain without tenderness and had no edema in the extremities.

His blood test findings are shown in Table 1. The Child–Pugh score was 9 points (B) with moderate or greater ascites volume and no encephalopathy. His platelet count was low. No renal impairment was observed.

**Table 1:** The patient’s laboratory data on admission

*Biochemistry*		*Hematology*	
TP	6.8 mg/dL	WBC	1,800/μL
ALB	2.4 mg/dL	RBC	357 × 10^4^/μL
T-bil	1.30 mg/dL	Hb	12.2 g/dL
D-bil	0.35 mg/dL	Plt	4.0 × 10^4^/μL
AST	64 IU/L	*Virus marker*	
ALT	60 IU/L	HBs Ag	(−)
ALP	311 IU/L	HCV Ab	(+)
γ-GTP	28 IU/L	*Urine examination*	
BUN	11.0 mg/dL	Specific gravity	1.010
Cre	0.81 mg/dL	pH	6.5
Na	144 mEq/L	Protein	(−)
K	2.9 mEq/L	Glucose	(−)
Cl	108 mEq/L	Occult blood	(−)
eGFR	71 mL/min/1.73 m^2^	Ketone	(−)
*Coagulation*		Urobilinogen	Normal
PT	70.3 %	Bilirubin	(−)

*TP* total protein, *ALB* albumin, *T-bil* total bilirubin, *D-bil* direct bilirubin, *AST* aspartate aminotransferase, *ALT* alanine aminotransferase, *ALP* alkaline phosphatase, *γ-GTP* gamma-glutamyl transpeptidase, *BUN* blood urea nitrogen, *Cre* creatinine, *Na* sodium, *K* potassium, *Cl* chloride, *eGFR* estimated glomerular filtration rate, *PT* prothrombin time, *WBC* white blood cells, *RBC* red blood cells, *Hb* hemoglobin, *Plt* platelets, *HBs Ag* hepatitis B core antigen, *HCV Ab* hepatitis C virus core antigen

**Fig. 1:**
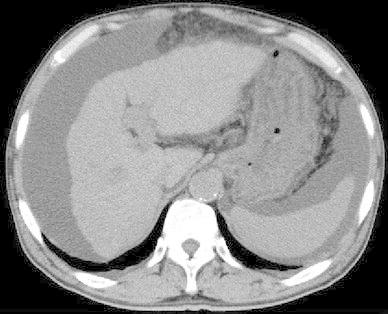
Plain abdominal computed tomography image. The hepatic lobes are atrophic with a blunt margin and irregularity on the surface. A moderate volume of ascites retention is also observed

On plain abdominal computed tomography (Fig. 1), both the hepatic lobes were atrophic with a blunt margin and irregularity on the surface. A moderate volume of ascites retention was observed.

The ascites fluid obtained following puncture indicated the presence of transudative ascites, which was macroscopically pale yellow and transparent with a cell count of 200/µL and a protein level of 2.0 g/dL. The cytological diagnosis was class I, and the culture test was negative.

**Fig. 2:**
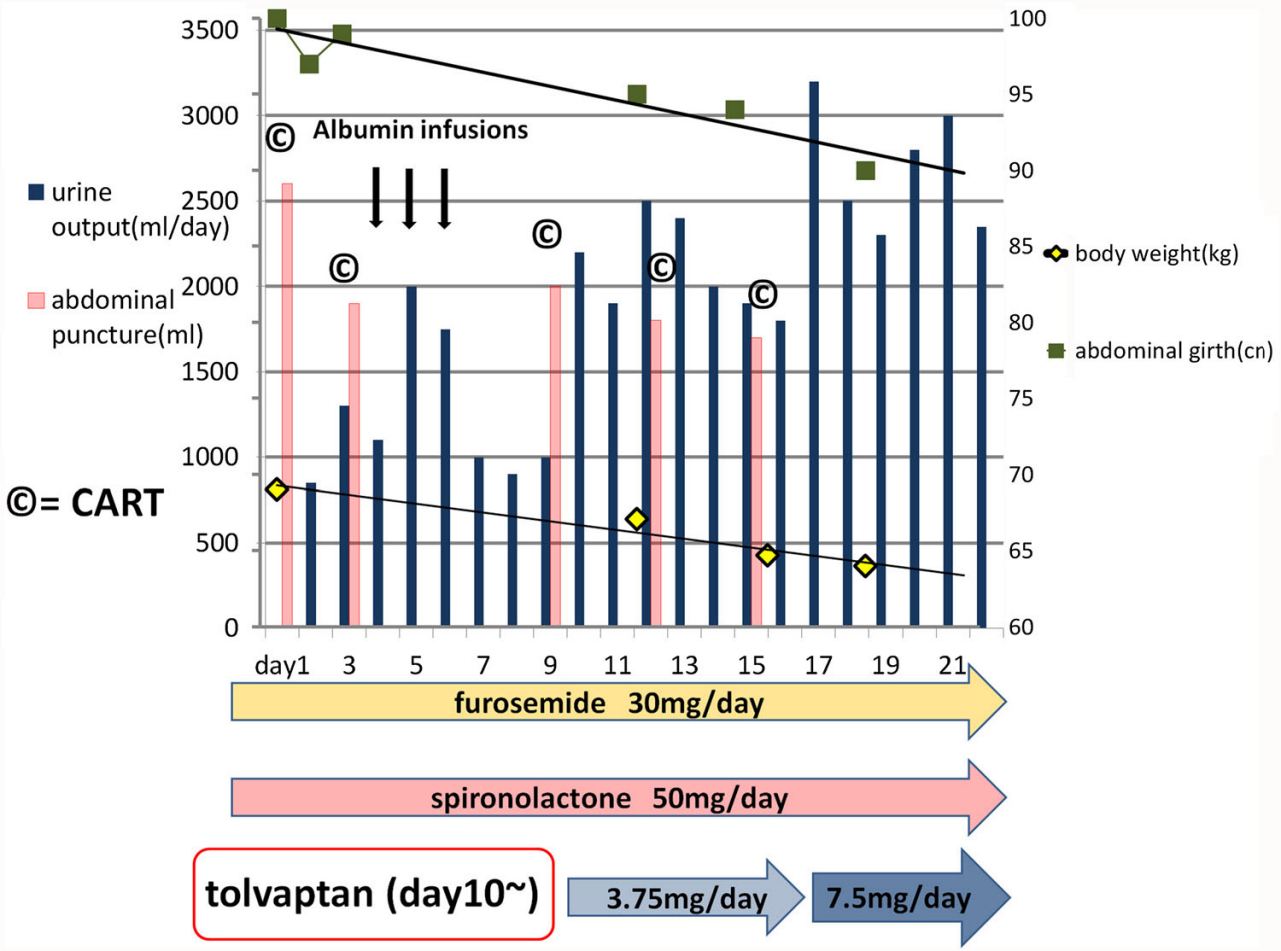
The patient’s clinical course after the first admission

The patient’s clinical course is presented in Fig. 2. The patient was on salt restriction (≤5 g/day) and water restriction (≤1 L/day), and he underwent three courses of cell-free and concentrated ascites reinfusion therapy (CART). However, he again exhibited ascites retention after several days, and the urinary volume was insufficient at 1,000–1,500 mL/day. On day 10, oral tolvaptan (3.75 mg/day) was started, and his urinary volume immediately increased to 2,000 mL/day. Adverse reactions, such as hypernatremia and liver dysfunction, were not observed. Since the patient still required puncture for ascites removal, the dose of tolvaptan was increased to 7.5 mg/day on day 17. His urinary volume increased to approximately 2,500 mL/day, and his body weight and abdominal circumference improved gradually without any puncture for ascites removal. His abdominal pain and bloating improved, and he was discharged on day 22.

**Fig. 3:**
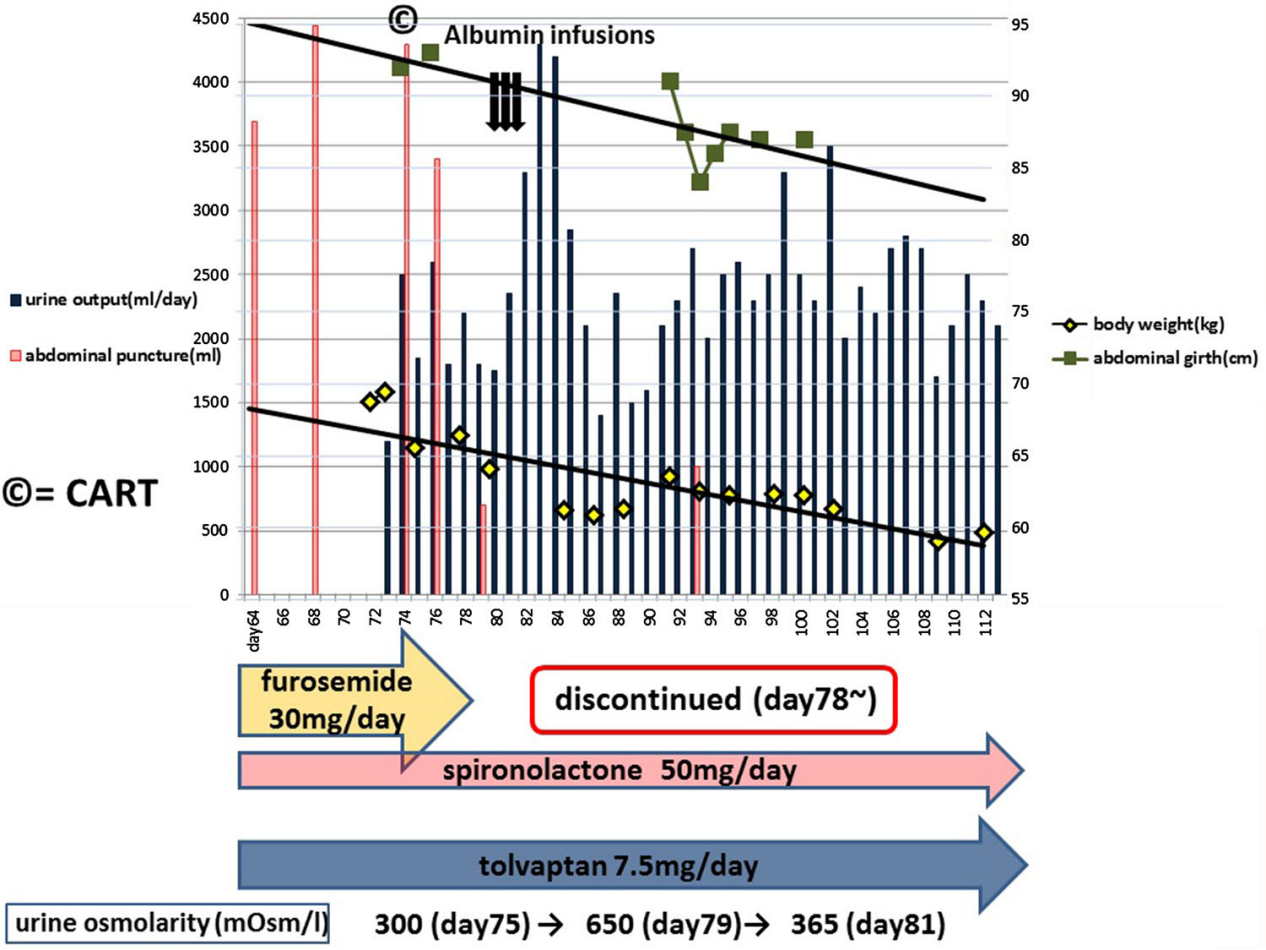
The patient’s clinical course after the second admission

He continued tolvaptan (7.5 mg/day) treatment, and at approximately day 60, the patient realized that his urinary volume had decreased. He presented with abdominal bloating and weight gain again. Re-exacerbation of the ascites was suspected, and the patient was readmitted on day 72 (Fig. 3). A sufficient urinary volume was not observed after admission. We presumed that urine osmolality decreased due to the continuous administration of furosemide, which may have hampered the diuretic effect of tolvaptan in the renal collecting tubule; furosemide was discontinued on day 78. Subsequently, his urinary volume temporarily increased to more than 4,000 mL/day. There is a possibility that the albumin infusions (days 78–80) affected the temporary increase in his urinary volume; however, a volume of ≥2,000 mL/day was maintained over the next month after discontinuing furosemide. The urine osmolality immediately increased from 300 to 650 mOsm/L after discontinuing furosemide. In addition, 3 days after discontinuing furosemide (day 81), the urine osmolality decreased to 365 mOsm/L. The abdominal symptoms, body weight, and abdominal circumference returned to their original levels. Tolvaptan was continued (7.5 mg/day) without exacerbation of the ascites. The serum sodium level was maintained (range 139–144 mEq/L) throughout the course.

## Discussion

The use of existing natriuretic agents for treating ascites associated with liver cirrhosis is problematic for the following reasons: hyponatremia is likely to occur; diuresis resistance or impaired renal function is induced by the activation of the renin–angiotensin–aldosterone system; and hypoalbuminemia-associated treatment resistance may occur. Tolvaptan acts on the renal collecting tubule, inhibits the reabsorption of water, and accelerates urinary excretion [1]. Since it results in the excretion of only free water without inducing natriuresis, tolvaptan improves hyponatremia [2]. Moreover, it does not affect the activation of the renin–angiotensin–aldosterone system, thereby allowing the body fluid to be controlled without impairing renal function [3]. According to Japanese and overseas large-scale clinical studies, it exhibits a diuretic effect and improves clinical symptoms in heart failure patients [4, 5].

Thuluvath et al. [6] reported that the administration of a vasopressin V2-receptor antagonist in addition to conventional treatment with furosemide and spironolactone increased urinary volume in liver cirrhosis patients. Okita et al. [7] first reported the results of a randomized, double-blind, placebo-controlled study on tolvaptan for refractory ascites associated with liver cirrhosis. When tolvaptan was administered to patients with hepatic edema at dosages of 30, 15, or 7.5 mg/day for 7 days, the maximum decrease in weight and abdominal circumference was observed in patients receiving 7.5 mg/day, thus providing the basis for the current upper dose limit in the present case. Sakaida et al. [8] reported that the weight loss obtained by short-term treatment with diuretics in liver cirrhosis patients strongly correlated with the volume of ascites, accounting for approximately 30 % of cases with weight loss. Moreover, in a randomized, double-blind, placebo-controlled study, they reported that a significant weight loss was obtained in patients receiving 7.5 mg/day of tolvaptan, revealing that it is effective for improving ascites [9]. They also reported that it is useful for treating patients with hypoalbuminemia (serum albumin level <2.5 g/dL), because of tolvaptan’s effect on the renal collecting tubule through the blood flow.

Although tolvaptan is expected to play an innovative role in treating refractory patients, no diuresis is achieved even after administration, and the efficacy of this drug can often be diminished, as seen in the present case. An approach to determine its appropriate use involves the elucidation of the responders to this drug. One possible approach is to use urine osmolality as a marker reflecting the function and activity of the renal collecting tubule. Imamura et al. [10] defined patients with heart failure who had an increase in urinary volume during 24 h after the start of tolvaptan administration as responders, revealing that responders showed a significant improvement in body weight, brain natriuretic peptide levels, and clinical symptoms. Moreover, they reported that the predictive factors of responders are urine osmolality of ≥352 mOsm/L before tolvaptan administration or a ≥26 % urine osmolality reduction at 4–6 h after administration [11–13]. The mechanism of water reabsorption by vasopressin is as follows: the binding of vasopressin to V2-receptors in the renal collecting tubule induces cyclic adenosine monophosphate dependent synthesis of the aquaporin-2 water channel and its migration to the cellular surface, which in turn leads to increased water permeability in the collecting tubule, thus resulting in enhanced water reabsorption mediated by an osmotic pressure gradient formed between the hypotonic urine in the collecting tubule and the hypertonic renal interstitium [14]. Tolvaptan competitively inhibits V2-receptor binding, thereby suppressing the expression of aquaporin-2 and inducing water diuresis. Patients who initially have a low urine osmolality may have a decreased expression of aquaporin-2 or a reduced osmotic pressure gradient, thus making it difficult for tolvaptan to be efficacious [11].

The urine osmolality increased from 300 to 650 mOsm/L after discontinuing furosemide in our patient. The urine osmolality should be evaluated before starting tolvaptan. Although the urine osmolality was originally at the level where an effective water diuretic effect could not be obtained, discontinuing furosemide may have increased urine osmolality in the medullary collecting tubule to the range where tolvaptan is effective, thereby achieving diuresis. In addition, 3 days after discontinuing furosemide, the urine osmolality decreased to 365 mOsm/L; thus, the urine osmolality may have decreased because of the sufficient water diuresis caused by tolvaptan. Since the maximum dose of tolvaptan has already been used, it is important to review its preparation by evaluating the osmolality of concomitant diuretic agents.

Our case is valuable, because a non-responder became a responder by adjusting a loop diuretic. The maximum dose of tolvaptan is 7.5 mg/day. However, if 7.5 mg of tolvaptan is administered and the patient’s urine volume decreases, the doctor may assume that this effect is a result of another diuretic. In our case, furosemide decreased the osmolality; therefore, we recommend that clinicians assess the osmolality and reconsider the decreasing effect of other diuretics. Similarly, Imamura et al. [15] reported that a patient with acute renal impairment did not respond to the initial administration but responded to tolvaptan when the renal impairment resolved and renal osmolality was increased. The diuretic effect of tolvaptan can be enhanced by considering the administration timing and by adjusting concomitant conventional diuretic agents. Although the appropriate dosing period for tolvaptan in patients with hepatic edema is unclear, there is a potential diminished effect after long-term administration. A future study on long-term users is needed to establish the efficacy of tolvaptan.
